# Supplementary material for: Treatment Resulting Changes in Volumes of High-18F-FDG-Uptake Adipose Tissues over Orbit and Epicardium Correlate with Treatment Response for Non-Hodgkin’s Lymphoma
Source: Int J Mol Sci. 2023 Jan 21;24(3):2158. doi: 10.3390/ijms24032158 (PMC9916748; doi:10.3390/ijms24032158)
Supplement: Supplementary file 1 [file ijms-24-02158-s001.zip › ijms-2132292-supplementary.pdf]

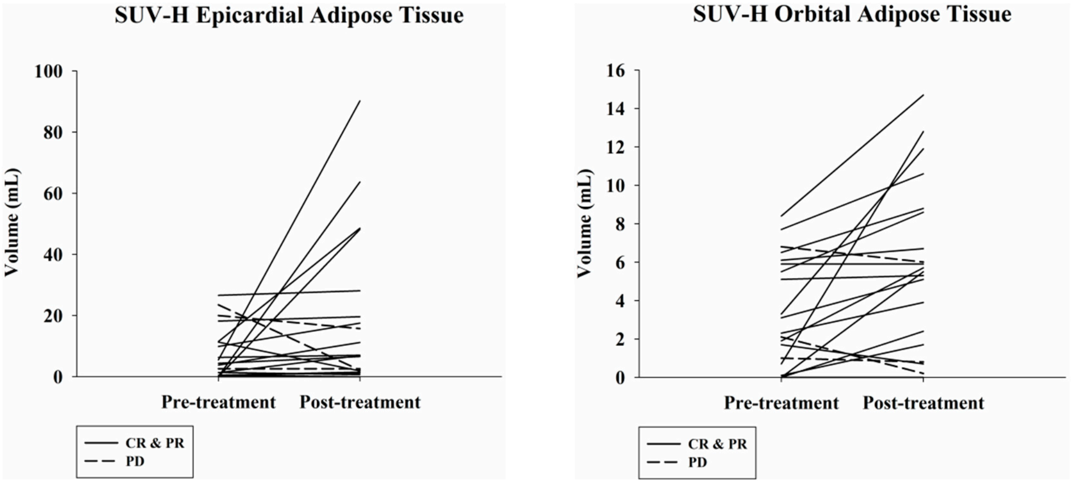

(**a**) (**b**)

Supplementary Figure S1. Each individual data point is plotted to illustrate the patient-specific local changes in (a) SUV-H EAT and (b) SUV-H OAT volume from pre- to post-treatment. SUV-H EAT, high standardized uptake value epicardial adipose tissue; SUV-H OAT, high standardized uptake value orbital adipose tissue; CR, complete response; PR, partial response; PD, progressive disease.

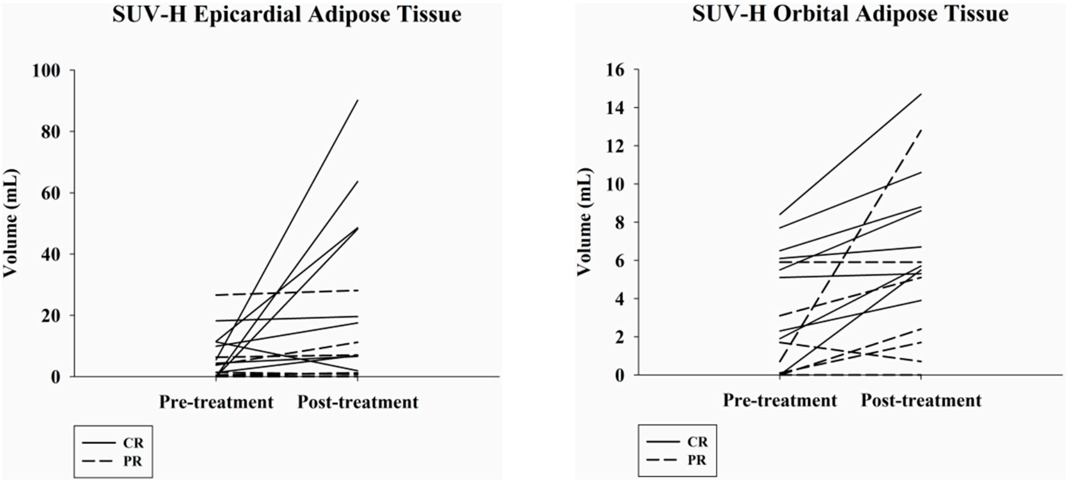

(**a**) (**b**)

Supplementary Figure S2. Each individual data point is plotted to illustrate the patient-specific local changes in (a) SUV-H EAT and (b) SUV-H OAT volume from pre- to post-treatment between complete and partial responders. SUV-H EAT, high standardized uptake value epicardial adipose tissue; SUV-H OAT, high standardized uptake value orbital adipose tissue; CR, complete response; PR, partial response.
